# Sociodemographic Characteristics, Comorbidities, and Mortality Among Persons Diagnosed With Tuberculosis and COVID-19 in Close Succession in California, 2020

**DOI:** 10.1001/jamanetworkopen.2021.36853

**Published:** 2021-12-03

**Authors:** Scott A. Nabity, Emily Han, Phil Lowenthal, Hannah Henry, Nnenna Okoye, Melony Chakrabarty, Amit S. Chitnis, Ankita Kadakia, Elsa Villarino, Julie Low, Julie Higashi, Pennan M. Barry, Seema Jain, Jennifer Flood

**Affiliations:** 1 California Department of Public Health, Richmond; 2 Centers for Disease Control and Prevention, Atlanta, Georgia; 3 Sacramento County Health Services, Sacramento, California; 4 Alameda County Public Health Department, San Leandro, California; 5 San Diego County Health and Human Services Agency, San Diego, California; 6 Santa Clara County Public Health Department, San Jose, California; 7 Orange County Health Care Agency, Santa Ana, California; 8 Los Angeles County Department of Health, Los Angeles, California

## Abstract

**Importance:**

Tuberculosis (TB) and COVID-19 are respiratory diseases that disproportionately occur among medically underserved populations; little is known about their epidemiologic intersection.

**Objective:**

To characterize persons diagnosed with TB and COVID-19 in California.

**Design, Setting, and Participants:**

This cross-sectional analysis of population-based public health surveillance data assessed the sociodemographic, clinical, and epidemiologic characteristics of California residents who were diagnosed with TB (including cases diagnosed and reported between September 3, 2019, and December 31, 2020) and COVID-19 (including confirmed cases based on positive results on polymerase chain reaction tests and probable cases based on positive results on antigen assays reported through February 2, 2021) in close succession compared with those who were diagnosed with TB before the COVID-19 pandemic (between January 1, 2017, and December 31, 2019) or diagnosed with COVID-19 alone (through February 2, 2021). This analysis included 3 402 713 California residents with COVID-19 alone, 6280 with TB before the pandemic, and 91 with confirmed or probable COVID-19 diagnosed within 120 days of a TB diagnosis (ie, TB/COVID-19).

**Exposures:**

Sociodemographic characteristics, medical risk factors, factors associated with TB severity, and health equity index.

**Main Outcomes and Measures:**

Frequency of reported successive TB and COVID-19 (TB/COVID-19) diagnoses within 120 days, frequency of deaths, and age-adjusted mortality rates.

**Results:**

Among the 91 persons with TB/COVID-19, the median age was 58.0 years (range, 3.0-95.0 years; IQR, 41.0-73.0 years); 52 persons (57.1%) were male; 81 (89.0%) were born outside the US; and 28 (30.8%) were Asian or Pacific Islander, 4 (4.4%) were Black, 55 (60.4%) were Hispanic or Latino, 4 (4.4%) were White. The frequency of reported COVID-19 among those who received a TB diagnosis between September 3, 2019, and December 31, 2020, was 225 of 2210 persons (10.2%), which was similar to that of the general population (3 402 804 of 39 538 223 persons [8.6%]). Compared with persons with TB before the pandemic, those with TB/COVID-19 were more likely to be Hispanic or Latino (2285 of 6279 persons [36.4%; 95% CI, 35.2%-37.6%] vs 55 of 91 persons [60.4%; 95% CI, 49.6%-70.5%], respectively; *P* < .001), reside in low health equity census tracts (1984 of 6027 persons [32.9%; 95% CI, 31.7%-34.1%] vs 40 of 89 persons [44.9%; 95% CI, 34.4%-55.9%]; *P* = .003), live in the US longer before receiving a TB diagnosis (median, 19.7 years [IQR, 7.2-32.3 years] vs 23.1 years [IQR, 15.2-31.5 years]; *P* = .03), and have diabetes (1734 of 6280 persons [27.6%; 95% CI, 26.5%-28.7%] vs 42 of 91 persons [46.2%; 95% CI, 35.6%-56.9%]; *P* < .001). The frequency of deaths among those with TB/COVID-19 successively diagnosed within 30 days (8 of 34 persons [23.5%; 95% CI, 10.8%-41.2%]) was more than twice that of persons with TB before the pandemic (631 of 5545 persons [11.4%; 95% CI, 10.6%-12.2%]; *P* = .05) and 20 times that of persons with COVID-19 alone (42 171 of 3 402 713 persons [1.2%; 95% CI, 1.2%-1.3%]; *P* < .001). Persons with TB/COVID-19 who died were older (median, 81.0 years; IQR, 75.0-85.0 years) than those who survived (median, 54.0 years; IQR, 37.5-68.5 years; *P* < .001). The age-adjusted mortality rate remained higher among persons with TB/COVID-19 (74.2 deaths per 1000 persons; 95% CI, 26.2-122.1 deaths per 1000 persons) compared with either disease alone (TB before the pandemic: 56.3 deaths per 1000 persons [95% CI, 51.2-61.4 deaths per 1000 persons]; COVID-19 only: 17.1 deaths per 1000 persons [95% CI, 16.9-17.2 deaths per 1000 persons]).

**Conclusions and Relevance:**

In this cross-sectional analysis, TB/COVID-19 was disproportionately diagnosed among California residents who were Hispanic or Latino, had diabetes, or were living in low health equity census tracts. These results suggest that tuberculosis and COVID-19 occurring together may be associated with increases in mortality compared with either disease alone, especially among older adults. Addressing health inequities and integrating prevention efforts could avert the occurrence of concurrent COVID-19 and TB and potentially reduce deaths.

## Key Points

**Question:**

What are the sociodemographic, clinical, and epidemiologic characteristics of persons diagnosed with tuberculosis (TB) and COVID-19 in close succession in California?

**Findings:**

In this cross-sectional analysis of public health surveillance records from California residents, 91 individuals diagnosed with TB and COVID-19 more commonly had Hispanic or Latino ethnicity, diabetes, and residence in a low health equity census tract compared with those who received a TB diagnosis before the COVID-19 pandemic. Mortality rates among those diagnosed with TB and COVID-19 in close succession were higher than mortality rates among those with TB before the COVID-19 pandemic and those with COVID-19 alone.

**Meaning:**

The findings of this analysis suggest that addressing long-standing health inequities and integrating prevention measures for COVID-19 and TB in California may reduce the co-occurrence of these diseases and prevent deaths.

## Teaser

This cross-sectional analysis uses surveillance data to examine clinical and epidemiologic characteristics of California residents diagnosed with tuberculosis and COVID-19 in close succession compared with those diagnosed with tuberculosis before the COVID-19 pandemic or COVID-19 alone.

## Introduction

The US has a low tuberculosis (TB) burden, and California, a diverse state with 40 million residents, reports one-quarter of the nation’s cases.^1^ Travelers to California were among the first to receive COVID-19 diagnoses in the US, producing subsequent widespread community transmission.^2^ Although TB and COVID-19 share some medical risk factors, little is known about the epidemiologic intersection of these primarily respiratory diseases, especially in settings with low TB incidence. We aimed to describe the epidemiologic characteristics and deaths associated with these diseases among California residents who were diagnosed with TB and COVID-19 in close succession in comparison with those among individuals diagnosed with TB before the pandemic.

## Methods

This cross-sectional analysis was approved by the California Department of Public Health and the Centers for Disease Control and Prevention. Because public health surveillance data were used for emergency response purposes, a nonresearch determination was made by both institutions, and informed consent was not required. The analysis followed the Strengthening the Reporting of Observational Studies in Epidemiology (STROBE) reporting guideline.

Diagnoses of persons with both TB and COVID-19 (TB/COVID-19) were defined using 2 criteria: (1) a diagnosis of TB and/or COVID-19 in California in 2020 and (2) successive diagnoses of TB and COVID-19 within 120 days. We selected the 120-day interval to represent persons who had acute illness with TB and COVID-19 in close succession. Although symptomatic COVID-19 has rapid onset and is typically diagnosed within days, TB generally has a slower onset, and diagnosis may take several weeks to months.

**Figure 1.**
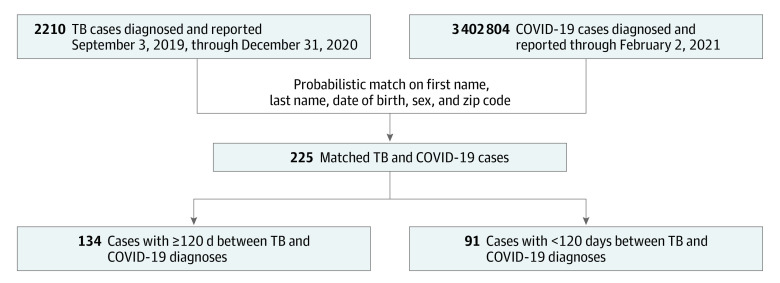
Analytic Sample Selection for Persons With Tuberculosis (TB) and COVID-19 Includes persons in California diagnosed with TB between September 3, 2019, and December 31, 2020, who were also diagnosed with COVID-19.

We first identified California residents with active TB who received a diagnosis between September 3, 2019 (ie, within 120 days of January 1, 2020), and December 31, 2020 (Figure 1). We then cross-matched these records with all confirmed (defined as a positive result on a polymerase chain reaction test) or probable (defined a positive result on an antigen assay) COVID-19 cases reported through February 2, 2021, the cutoff date when the match with TB cases was performed. We used surveillance records from the California Reportable Disease Information Exchange (CalREDIE), the communicable disease surveillance database maintained by the California Department of Public Health. We used a probabilistic algorithm to match first name, last name, date of birth, sex, and zip code in Match*Pro software, version 1.6.2 (National Cancer Institute), and required an exact match on at least one of first name, last name, or date of birth.

The TB diagnosis date was the earliest recorded among the report date, treatment start date, or specimen collection date during which a positive result on a blood culture or nucleic acid amplification analysis was recorded. We defined the COVID-19 diagnosis date as the earliest among specimen collection dates during which a positive test result was recorded. We considered positive results for cavitary disease (via chest radiography or computed tomography) and sputum smear tests for acid-fast bacilli to potentially indicate advanced pulmonary TB. Disseminated TB was defined as a positive blood culture result for *Mycobacterium tuberculosis* complex, meningeal involvement, miliary disease, or both extrapulmonary and pulmonary disease.

For persons diagnosed with TB before the COVID-19 pandemic (January 1, 2017, to December 31, 2019), we used TB surveillance records to ascertain deaths before or during TB treatment. In general, 174 TB cases were diagnosed per month from January 1, 2017, to December 31, 2019. From September 3, 2019, to December 31, 2020, approximately 147 TB cases were diagnosed per month.

For persons with TB/COVID-19 and persons with COVID-19 alone, we ascertained death status as of April 10, 2021, by using COVID-19 surveillance records and by cross-referencing reported COVID-19 cases in the California vital statistics database. Cases were assigned to quartiles according to scores on the California Healthy Places Index,^3^ a multidomain composite social inequity index, by census tract of residence (with quartile 1 indicating least advantaged and quartile 4 indicating most advantaged).

### Statistical Analysis

Matched persons with TB/COVID-19 were compared with all 6280 persons diagnosed with TB before the COVID-19 pandemic, with frequency of death included in comparisons. Using the same numerators, we directly age adjusted mortality rates per 1000 residents to the 2020 California Department of Finance standard population (10-year age strata flanked by the age groups of ≤14 years and ≥75 years).^4^ One person was diagnosed with TB in December 2019 and with COVID-19 in April 2020; this person was part of the TB/COVID-19 analysis group and excluded from the prepandemic TB analysis group. For statistical comparisons, we used 2-sided χ2 or Fisher exact tests for categorical data and Wilcoxon 2-sample tests for continuous data. Log-normal CIs for rate ratios were calculated using SAS software, version 9.4 (SAS Institute, Inc). The significance threshold was *P* = .05.

## Results

Of 3 409 084 total California residents included in the analysis, 3 402 713 persons had COVID-19 alone, 6280 had TB before the pandemic, and 91 had confirmed or probable COVID-19 diagnosed within 120 days of a TB diagnosis (ie, TB/COVID-19). Among those with TB/COVID-19, the median age was 58.0 years (range, 3.0-95.0 years; IQR, 41.0-73.0 years); 52 persons (57.1%) were male, 39 (42.9%) were female, and 81 (89.0%) were born outside the US (Table). A total of 28 persons (30.8%) were Asian or Pacific Islander, 4 (4.4%) were Black, 55 (60.4%) were Hispanic or Latino, 4 (4.4%) were White, and 0 were of other races or ethnicities. The frequency of COVID-19 among persons with TB diagnosed between September 3, 2019, and December 31, 2020, was similar to that of the general population in California (225 of 2210 persons [10.2%] vs 3 402 804 of 39 538 223 persons [8.6%], respectively) (Figure 1). In contrast to those with TB before the COVID-19 pandemic, those with TB/COVID-19 were more frequently Hispanic or Latino (55 of 91 persons [60.4%; 95% CI, 49.6%-70.5%] vs 2285 of 6279 persons [36.4%; 95% CI, 35.2%-37.6%]; *P* < .001), less frequently Asian or Pacific Islander (28 of 91 persons [30.8%; 95% CI 21.5%-41.3%] vs 3323 of 6279 persons [52.9%; 95% CI, 51.7%-54.2%]; *P* < .001), and more frequently residing in low health equity census tracts (eg, Healthy Places Index quartile 1: 40 of 89 persons [44.9%; 95% CI, 34.4%-55.9%] vs 1984 of 6027 persons [32.9%; 95% CI, 31.7%-34.1%]; *P* = .003). Persons not born in the US who had TB/COVID-19 resided in the US longer before receiving their TB diagnosis than those with TB before the pandemic (median, 23.1 years [IQR, 15.2-31.5 years] vs 19.7 years [IQR, 7.2-32.3 years], respectively; *P* = .03).

**Table.** Characteristics of Persons Diagnosed With Tuberculosis (TB) and COVID-19 Within 120 Days vs Persons With TB Before COVID-19 Pandemic in California^a^

Characteristic	TB and COVID-19		TB before pandemic		*P* value
	No./total No.	% (95% CI)	No./total No.	% (95% CI)	
Participants, No.	91	NA	6280	NA	NA
Age, median (IQR), y	91/91	58.0 (41.0-73.0)	6280/6280	56.0 (35.0-70.0)	.16
Sex					
Female	39/91	42.9 (32.5-53.7)	2441/6280	38.9 (37.7-40.1)	.44
Male	52/91	57.1 (46.3-67.5)	3839/6280	61.1 (59.9-62.3)	
Race and/or ethnicity					
Asian or Pacific Islander	28/91	30.8 (21.5-41.3)	3323/6279	52.9 (51.7-54.2)	<.001
Black	4/91	4.4 (1.2-10.9)	288/6279	4.6 (4.1-5.1)	
Hispanic or Latino	55/91	60.4 (49.6-70.5)	2285/6279	36.4 (35.2-37.6)	
White	4/91	4.4 (1.2-10.9)	372/6279	5.9 (5.4-6.5)	
Other^b^	0	0	11/6279	0.2 (0.1-0.3)	
Not born in US	81/91	89.0 (80.7-94.6)	5158/6268	82.3 (81.3-83.2)	.09
Years in US before TB diagnosis, median (IQR)	76/81	23.1 (15.2-31.5)	4914/5158	19.7 (7.2-32.3)	.03
Healthy Places Index score quartile					
1 (least advantaged)	40/89	44.9 (34.4-55.9)	1984/6027	32.9 (31.7-34.1)	.003
2	29/89	32.6 (23.0-43.3)	1569/6027	26.0 (24.9-27.2)	
3	15/89	16.9 (9.8-26.3)	1378/6027	22.9 (21.8-24.0)	
4 (most advantaged)	5/89	5.6 (1.9-12.6)	1096/6027	18.2 (17.2-19.2)	
Previous TB disease	1/90	1.1 (0.0-6.0)	323/6276	5.1 (4.6-5.7)	.09
Site of TB disease					
Pulmonary only	65/91	71.4 (61.0-80.4)	4357/6278	69.4 (68.2-70.5)	.88
Nonpulmonary only	16/91	17.6 (10.4-27.0)	1129/6278	18.0 (17.0-19.0)	
Pulmonary and nonpulmonary	10/91	11.0 (5.4-19.3)	792/6278	12.6 (11.8-13.5)	
Positive sputum smear test result for pulmonary TB^c^	45/71	63.4 (51.1-74.5)	2515/4870	51.6 (50.2-53.1)	.05
Cavitary pulmonary disease^d^	33/74	44.6 (33.0-56.6)	1709/5133	33.3 (32.0-34.6)	.04
Disseminated TB disease^e^	13/91	14.3 (7.8-23.2)	977/6280	15.6 (14.7-16.5)	.74
Any initial isoniazid or rifampicin resistance	4/60	6.7 (1.9-16.2)	543/5136	10.6 (9.7-11.5)	.33
Medical risk factors					
Diabetes	42/91	46.2 (35.6-56.9)	1734/6280	27.6 (26.5-28.7)	<.001
HIV	4/82	4.9 (1.3-12.0)	197/5576	3.5 (3.1-4.1)	.54
End-stage kidney disease	5/91	5.5 (1.8-12.4)	250/6280	4.0 (3.5-4.5)	.41
Other immunocompromised condition^f^	7/91	7.7 (3.2-15.2)	471/6280	7.5 (6.9-8.2)	.94
Two or more medical risk factors	7/91	7.7 (3.2-15.2)	245/6280	3.9 (3.4-4.4)	.09
Occupation					
Health care worker	5/82	6.1 (2.0-13.7)	246/6162	4.0 (3.5-4.5)	.57
Correctional facility employee	0		1/6162	0 (0-0.1)	
Migrant or seasonal worker	2/82	2.4 (0.3-8.5)	125/6162	2.0 (1.7-2.4)	
Other occupation	21/82	25.6 (16.7-36.4)	2075/6162	33.7 (32.5-34.9)	
Not employed	54/82	65.9 (54.6-76.0)	3715/6162	60.3 (59.1-61.5)	
Social and behavioral characteristics					
Homeless	2/89	2.2 (0.3-7.9)	325/6256	5.2 (4.7-5.8)	.33
Correctional facility resident	2/91	2.2 (0.3-7.7)	158/6274	2.5 (2.1-2.9)	>.99
Long-term care facility resident	5/91	5.5 (1.8-12.4)	148/6271	2.4 (2.0-2.8)	.07
Substance misuse^g^	7/87	8.0 (3.3-15.9)	650/6138	10.6 (9.8-11.4)	.44
Deaths stratified by interval between TB and COVID-19 diagnoses^h^					
Within 120 d	15/91	16.5 (9.5-25.7)	631/5545	11.4 (10.6-12.2)	.13
Within 90 d	13/69	18.8 (10.4-30.1)	NA	NA	.05
Within 60 d	10/51	19.6 (9.8-33.1)	NA	NA	.07
Within 30 d	8/34	23.5 (10.8-41.2)	NA	NA	.05
Dead at TB diagnosis	2/91	2.2 (0.3-7.7)	119/6280	1.9 (1.6-2.3)	.69

Abbreviation: NA, not applicable.

^a^ Statistical comparisons were made using χ^2^ or Fisher exact tests for categorical data and the Wilcoxon 2-sample test for continuous data. The TB diagnosis date was defined as the earliest of report date, treatment start date, or specimen collection date during which a positive result on a culture or nucleic acid amplification analysis was recorded. The COVID-19 diagnosis date was defined as the earliest laboratory specimen collection date with a positive test result. Persons with TB/COVID-19 were those successively diagnosed with TB and COVID-19 within 120 days for whom at least one of the diseases was diagnosed in 2020. Persons with TB before the pandemic included those diagnosed with TB between January 1, 2017, and December 31, 2019. In general, 174 TB cases were diagnosed per month during this period. From September 3, 2019, to December 31, 2020, approximately 147 TB cases were diagnosed per month. Unknown and missing values were excluded from all subcategories.

^b^ Includes American Indian or Alaska Native (2 of 6279 persons [0.03%]) and multiple races or ethnicities (9 of 6279 persons [0.1%]).

^c^ Denominator does not sum to total number with pulmonary TB because not all persons with pulmonary TB received a sputum smear test for acid-fast bacilli.

^d^ Cavity identified on chest radiography or computed tomography among persons with pulmonary TB when imaging was performed.

^e^ Meningeal, miliary, positive result on acid-fast bacilli blood culture, or both pulmonary and extrapulmonary TB.

^f^ Includes persons receiving immunosuppressive therapies (eg, tumor necrosis factor α antagonist or high-dose steroid medications) and persons with medical conditions, such as hematological cancer.

^g^ Any illicit injectable or noninjectable drug use or excess alcohol consumption within the 12 months before TB diagnosis.

^h^ Statistical comparison was made between all prepandemic TB deaths (631 of 5545 persons [11.4%]) and TB/COVID-19 deaths within each interval between diagnoses.

Thirty-two persons (35.2%) with TB/COVID-19 were diagnosed with COVID-19 before they were diagnosed with TB, with a median of 17.5 days (IQR, 1.5-53.0 days) from COVID-19 diagnosis to TB diagnosis; 10 persons (11.0%) were successively diagnosed with TB and COVID-19 within 2 days. Diabetes was more frequent among persons with TB/COVID-19 than those with TB before the pandemic (42 of 91 persons [46.2%; 95% CI, 35.6%-56.9%] vs 1734 of 6280 persons [27.6%; 95% CI, 26.5%-28.7%], respectively; *P* < .001) (Table). A higher proportion of persons with pulmonary TB and COVID-19 vs persons with pulmonary TB before the pandemic had positive results on sputum smear testing (45 of 71 persons [63.4%; 95% CI, 51.1%-74.5%] vs 2515 of 4870 persons [51.6%; 95% CI, 50.2%-53.1%], respectively; *P* = .05) and lung cavities detected on chest imaging (33 of 74 persons [44.6%; 95% CI, 33.0%-56.6%] vs 1709 of 5133 persons [33.3%; 95% CI, 32.0%-34.6%]; *P* = .04). The frequency of positive results on sputum smear testing among persons who had pulmonary TB without COVID-19 in 2020 (50.3%) was similar to that of previous years (50.5% in 2016, 50.1% in 2017, 47.8% in 2018, and 48.8% in 2019), as was the proportion of persons with lung cavities detected on chest radiography (20.1% in 2020 vs 18.2% in 2016, 20.9% in 2017, 19.9% in 2018, and 19.4% in 2019) or computed tomographic scans (41.9% in 2020 vs 39.7% in 2016, 39.3% in 2017, 41.1% in 2018, and 38.4% in 2019).

**Figure 2.**
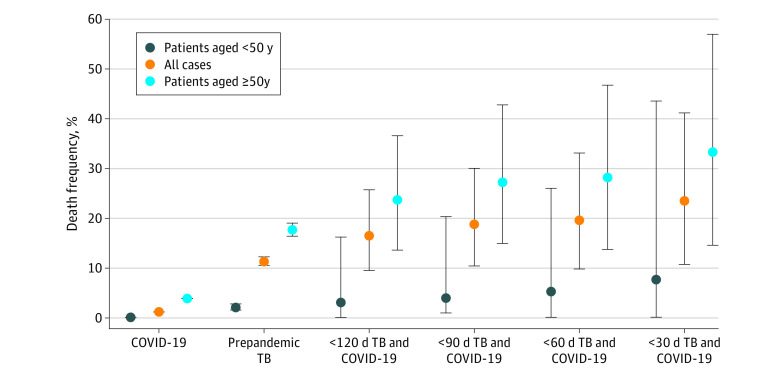
Age-Stratified Frequency of Death Frequency of death among California residents with COVID-19, tuberculosis (TB) before the COVID-19 pandemic, and both diseases successively diagnosed within 30, 60, 90, and 120 days in 2020. The frequency of death among persons with COVID-19 was calculated as 42 171 deaths among 3 402 713 persons diagnosed with COVID-19 as of February 2, 2021. Error bars represent Fisher exact 95% CIs for proportions. Tuberculosis cases were diagnosed and reported between September 3, 2019, and December 31, 2020. Persons with TB and COVID-19 included those with TB diagnoses reported through December 31, 2020, and COVID-19 diagnoses reported through February 2, 2021. Tuberculosis cases before the COVID-19 pandemic were reported from January 1, 2017, to December 31, 2019. One person was diagnosed with TB in December 2019 and COVID-19 in April 2020; this person was not included in the TB analysis group before the COVID-19 pandemic.

The frequency of death increased as the time between TB and COVID-19 diagnoses decreased (eg, 15 deaths among 91 persons [16.5%] at <120 days between diagnoses vs 8 deaths among 34 persons [23.5%] at <30 days between diagnoses), and deaths were higher among those 50 years and older (eg, persons with TB/COVID-19 with <120 days between diagnoses: 14 deaths among 59 persons [23.7%] aged ≥50 years vs 1 death among 32 persons [3.1%] aged <50 years) (Figure 2). Among persons with successive diagnoses within 30 days, the frequency of death among those with TB/COVID-19 (8 of 34 persons [23.5%; 95% CI, 10.8%-41.2%]) was more than twice that of persons with TB before the pandemic (631 of 5545 persons [11.4%; 95% CI, 10.6%-12.2%]; *P* = .05) and 20 times that of persons with COVID-19 alone (42 171 of 3 402 713 persons [1.2%; 95% CI, 1.2%-1.3%]; *P* < .001) (Figure 2). The 15 persons with TB/COVID-19 who died were older (median age, 81.0 years; IQR, 75.0-85.0 years; *P* < .001) than the 76 persons with TB/COVID-19 who survived (median, 54.0 years; IQR, 37.5-68.5 years; *P* < .001). Most deaths among persons with TB/COVID-19 (9 of 15 persons [60.0%]) occurred in those who had diabetes or another immunocompromising condition, which included HIV, end-stage kidney disease, hematological cancer, or immunosuppressive therapies. After adjustment for age, the mortality rate for persons with TB/COVID-19 (74.2 deaths per 1000 persons; 95% CI, 26.2-122.1 deaths per 1000 persons) remained 1.3 times higher (95% CI, 0.7-2.5) than that of persons with TB before the COVID-19 pandemic (56.3 deaths per 1000 persons; 95% CI, 51.2-61.4 deaths per 1000 persons) and 4.4 times higher (95% CI, 2.3-8.3) than that of persons with COVID-19 alone (17.1 deaths per 1000 persons; 95% CI, 16.9-17.2 deaths per 1000 persons) during the observation period.

## Discussion

This cross-sectional analysis is, to our knowledge, one of the first population-based analyses of TB and COVID-19 surveillance data in a low-incidence setting for TB. We found that California residents with TB/COVID-19 had characteristics that were generally similar to those of persons with TB before the COVID-19 pandemic. The frequency of COVID-19 among persons with TB (10.2%) approximated the frequency reported for COVID-19 in the general population at the time of this analysis (8.6%).^5^ Nevertheless, we identified characteristics that were more common among persons with TB/COVID-19, including Hispanic or Latino ethnicity, the presence of diabetes, and residence in low health equity census tracts, which reflected COVID-19 disparities in California.^6^ Similar to observations in other epidemiologic setting,^7,8,9,10^ deaths were more frequent among persons diagnosed with TB/COVID-19 and predominately occurred among older adults. When adjusted for age, the mortality rate for persons with TB/COVID-19 remained higher than that of persons with either disease alone. Plausible mechanisms for increased mortality include impaired pulmonary reserve resulting from preexisting TB, delayed TB diagnosis and health system disruption,^1^ and synergistic immune dysregulation among those with SARS-CoV-2 and *M tuberculosis* complex coinfection.^11,12^ Overlapping clinical risk factors for TB and COVID-19 could also have been associated with the worse outcomes observed among persons with TB/COVID-19. The increased frequency of positive results on sputum smear testing and cavitary disease among persons with TB/COVID-19 might reflect delays in TB diagnoses.

### Limitations

This analysis has limitations. We were unable to measure certain clinical factors associated with disease severity, such as hospitalization, or evaluate the circumstances associated with deaths. Whereas TB surveillance data were generally complete, a high frequency of missing sociodemographic data in the COVID-19 surveillance database precluded direct case-level comparisons between persons with TB/COVID-19 and persons with COVID-19 alone in 2020. The small number of persons with TB/COVID-19 produced wide ranges in CIs for rate comparisons. Nevertheless, we likely underestimated mortality among persons with TB/COVID-19 because follow-up surveillance reporting for TB, which captures death at any point during TB treatment, is not yet complete for 2020. In addition, this lack of follow-up data did not allow comparison of outcomes among persons diagnosed with TB in 2020 who did not have COVID-19. Additional reasons we may have underestimated mortality include the possibility that persons who survived their initial illness could have survived for up to 120 days to allow for the second diagnosis, and COVID-19–associated deaths peaked in California after our observation period.^5^ Underdetection of both COVID-19 and TB may have occurred in 2020, and the consequences this underdetection may have had for our surveillance results are an important area of future inquiry.

## Conclusions

This cross-sectional analysis found that during the first year of the COVID-19 pandemic, California residents with TB/COVID-19 had higher mortality than those with either disease alone; however, additional studies in the US are needed to assess the generalizability of these findings. Tuberculosis disproportionately occurs in medically and socially vulnerable communities, and these results suggest potential benefit from the integration of TB and COVID-19 prevention efforts, such as combining COVID-19 vaccination outreach with targeted screening for TB. Reductions in reported TB during the pandemic likely reflected, in part, decreased TB detection in the US.^1^ These reductions highlight the need for health care professionals to consider TB as a potential diagnosis among persons at risk and in the appropriate clinical context, including the possibility of *M tuberculosis* coinfection among persons with positive test results for SARS-CoV-2.

## References

[1] Deutsch-Feldman M, Pratt RH, Price SF, Tsang CA, Self JL. Tuberculosis—United States, 2020. MMWR Morb Mortal Wkly Rep. 2021;70(12):409-414. doi:10.15585/mmwr.mm7012a1 33764959 PMC7993554

[2] Jorden MA, Rudman SL, Villarino E, ; CDC COVID-19 Response Team. Evidence for limited early spread of COVID-19 within the United States, January–February 2020. MMWR Morb Mortal Wkly Rep. 2020;69(22):680-684. doi:10.15585/mmwr.mm6922e1 32497028 PMC7315848

[3] Public Health Alliance of Southern California. The California Healthy Places Index. Public Health Alliance of Southern California; 2018. Accessed March 12, 2021. http://healthyplacesindex.org

[4] State of California Department of Finance. Report P-2C: population projections by sex and 5-year age group, 2010-2060: California counties (2019 baseline). State of California Department of Finance; 2021. Accessed May 12, 2021. https://www.dof.ca.gov/Forecasting/Demographics/Projections/documents/P2C_County_Age-Group_Sex.xlsx

[5] State of California. Tracking COVID-19 in California. State of California; 2021. Accessed May 12, 2021. https://covid19.ca.gov/state-dashboard/

[6] Reitsma MB, Claypool AL, Vargo J, . Racial/ethnic disparities in COVID-19 exposure risk, testing, and cases at the subcounty level In California. *Health Aff (Millwood)*. 2021:40(6):870-878. doi:10.1377/hlthaff.2021.00098 PMC8458028 33979192

[7] Western Cape Department of Health; National Institute for Communicable Diseases, South Africa. Risk factors for coronavirus disease 2019 (COVID-19) death in a population cohort study from the Western Cape Province, South Africa. *Clin Infect Dis*. 2021;73(7):e2005-e2015. doi:10.1093/cid/ciaa1198 PMC7499501 32860699

[8] Tadolini M, Codecasa LR, Garcia-Garcia JM, . Active tuberculosis, sequelae and COVID-19 co-infection: first cohort of 49 cases. Eur Respir J. 2020;56(1):2001398. doi:10.1183/13993003.01398-2020 32457198 PMC7251245

[9] Sy KTL, Haw NJL, Uy J. Previous and active tuberculosis increases risk of death and prolongs recovery in patients with COVID-19. Infect Dis (Lond). 2020;52(12):902-907. doi:10.1080/23744235.2020.1806353 32808838

[10] Kumar MS, Surendran D, Manu MS, Rakesh PS, Balakrishnan S. Mortality due to TB–COVID-19 coinfection in India. Int J Tuberc Lung Dis. 2021;25(3):250-251. doi:10.5588/ijtld.20.0947 33688819

[11] Visca D, Ong CWM, Tiberi S, . Tuberculosis and COVID-19 interaction: a review of biological, clinical and public health effects. Pulmonology. 2021;27(2):151-165. doi:10.1016/j.pulmoe.2020.12.012 33547029 PMC7825946

[12] Riou C, du Bruyn E, Stek C, ; HIATUS Consortium. Relationship of SARS-CoV-2–specific CD4 response to COVID-19 severity and impact of HIV-1 and tuberculosis coinfection. J Clin Invest. 2021;131(12):e149125. doi:10.1172/JCI149125 33945513 PMC8203446

